# Serotypes, MIC-Based Antimicrobial Susceptibility, and Genotypic Diversity of *Actinobacillus pleuropneumoniae* Isolates from Diseased Pigs in Brazil

**DOI:** 10.3390/microorganisms14040828

**Published:** 2026-04-04

**Authors:** Barbara L. P. Costa, Carlos E. C. Matajira, André P. Poor, Matheus S. Monteiro, Luisa Z. Moreno, Maurício C. Dutra, Andrea M. Moreno

**Affiliations:** 1 Department of Preventive Veterinary Medicine and Animal Health, School of Veterinary Medicine and Animal Science, University of São Paulo, Av. Prof. Dr. Orlando Marques de Paiva, 87, Cidade Universitária, Sao Paulo 05508-270, SP, Brazilmatheus.salibamonteiro@gmail.com (M.S.M.); luisa.moreno@unesp.br (L.Z.M.);; 2 Laboratory of Microbial Biomolecules, School of Pharmaceutical Sciences, University of São Paulo, São Paulo 05508-900, SP, Brazil; 3 Department of Pathology, Reproduction, and One Health (DPRSU), School of Agricultural and Veterinary Studies (FCAV), São Paulo State University (UNESP), Jaboticabal 01049-010, SP, Brazil; 4 Consultoria GFD, Campinas 13026-515, SP, Brazil

**Keywords:** *Actinobacillus pleuropneumoniae*, serotyping, genotyping, antimicrobial resistance

## Abstract

*Actinobacillus pleuropneumoniae* is a major swine pathogen that causes pleuropneumonia and leads to substantial economic losses due to mortality, impaired growth, and carcass condemnation. Nineteen serovars have been described, and their geographic distribution has been assessed using multiple typing approaches. High serovar diversity, together with limited cross-protective immunity, increases reliance on antimicrobial therapy for disease control. However, data on the genotypic diversity and antimicrobial susceptibility of *A. pleuropneumoniae* remain limited worldwide, and information on serovar distribution in Brazil is scarce. Here, we report serotyping, genotyping, and antimicrobial susceptibility profiling of *A. pleuropneumoniae* isolated from diseased pigs in Brazil. Eighty-five isolates from eight Brazilian states were analyzed; serovars 5 and 10 were the most prevalent (38.8% and 29.4%, respectively). Ceftiofur, spectinomycin, gentamicin, neomycin, tilmicosin, tulathromycin, and florfenicol showed good in vitro activity against the isolates. The highest resistance rates were observed for tylosin (98.8%), clindamycin (90.6%), chlortetracycline (67.1%), and oxytetracycline (67.1%), and multidrug resistance was detected in 55% of strains. SE-AFLP and PFGE revealed high genetic diversity, including among isolates of the same serovar, although a modest tendency to cluster by geographic origin and serovar was observed.

## 1. Introduction

*Actinobacillus pleuropneumoniae* is one of the bacteria most frequently isolated from swine respiratory infections and is the etiologic agent of pleuropneumonia [1,2]. The clinical course can be peracute, acute, or chronic, depending on factors such as the initial infectious dose, serotype, and toxin profile, as well as the age and immune status of the host. *A. pleuropneumoniae* can infect pigs of all ages; however, its epidemiology is closely associated with the production stage. Piglets are partially protected during the first weeks of life by maternal antibodies, but susceptibility increases after weaning as this passive immunity declines. Although the disease may occur in other production groups, including breeding animals [1,3], clinical cases are most frequently observed in growing–finishing pigs, in which the highest incidence of outbreaks has been reported [2]. In peracute and acute forms, high morbidity and mortality may occur [4], which can contribute to increased sow mortality in some Brazilian herds [5]. Infection reduces herd profitability by increasing mortality, worsening feed conversion, and raising treatment costs, and reducing average daily gain by up to 33.6% [4,6,7]. Furthermore, fibrinous pleuritis lesions increase carcass condemnation [8].

Nineteen serotypes have been described based on surface carbohydrates, mainly capsular polysaccharides, and their prevalence varies among countries and over time [1,9]. In addition, cross-protection mediated by antibodies against different serotypes is limited, which can make commercial vaccines ineffective in some settings [1].

Serotype characterization alone may fail to elucidate the genetic diversity and infection dynamics of *A. pleuropneumoniae* [10]. Fingerprinting techniques provide better discrimination among closely related organisms at the species and strain levels and are valuable tools for differentiating *A. pleuropneumoniae* strains [11].

Another key issue in controlling *A. pleuropneumoniae* infection is the growing concern about antimicrobial resistance. Increasing resistance rates over the years have been reported [12], and 33% of isolates have been described as resistant to more than three antimicrobial classes (multidrug resistance [MDR]) [13,14]. High resistance rates have been observed for sulfonamides, tetracyclines, penicillins, and third-generation quinolones [12,13,14,15,16]. Despite the substantial health and economic impact of this pathogen, few studies have evaluated the minimum inhibitory concentrations (MICs) of antimicrobials against *A. pleuropneumoniae* [14,16], and only a limited number have assessed Brazilian strains [17,18]. Moreover, longitudinal interpretation of resistance trends depends on the availability of robust baseline datasets from earlier periods.

Therefore, the aim of this study was to establish a descriptive historical and epidemiological baseline for *A. pleuropneumoniae* in Brazil using molecular epidemiology data, focusing on the main serotypes circulating in each region and their associations with antimicrobial resistance profiles.

## 2. Materials and Methods

### 2.1. Bacterial Isolation

Eighty-five *A. pleuropneumoniae* strains were isolated from lung samples, collected at necropsy from naturally infected pigs from herds with a clinical history of pleuropneumonia (2006–2013). The animals originated from eight Brazilian states: Goiás (GO), Minas Gerais (MG), Mato Grosso (MT), Mato Grosso do Sul (MS), Paraná (PR), Rio Grande do Sul (RS), Santa Catarina (SC), and São Paulo (SP). The strains belonged to the culture collection of the Laboratory of Swine Health, Department of Preventive Veterinary Medicine and Animal Health (VPS), School of Veterinary Medicine and Animal Science, University of São Paulo (FMVZ-USP) (São Paulo, Brazil). After isolation, strains were stored at −80 °C in broth containing skim milk (Difco, Sparks, MD, USA) and glycerol until further analysis.

### 2.2. Culture Conditions

For reactivation, frozen stocks were thawed and inoculated into 4 mL of Brain Heart Infusion (BHI) broth (Difco, Sparks, MD, USA) supplemented with nicotinamide adenine dinucleotide (NAD) (Sigma, St. Louis, MO, USA) and 5% fetal bovine serum (LGC Biotecnologia, Cotia, SP, Brazil). Culture purity was assessed by plating onto Columbia agar (Difco, Sparks, MD, USA) supplemented with 5% sheep blood and cross-streaking with *Staphylococcus aureus* ATCC 25923 as an NAD source. Tubes and plates were incubated under microaerophilic conditions at 37 °C for 24 h. After confirming purity and colony morphology, 1 mL of the BHI culture was aliquoted for DNA extraction.

### 2.3. Molecular Identification and Serotyping

Genomic DNA was extracted according to the protocol described by Boom et al. [19]. Species identification of *A. pleuropneumoniae* was confirmed by polymerase chain reaction (PCR) using the primers and conditions described by Gram and Ahrens [20]. Molecular serotyping was performed using the protocols and primers described by Bossé et al. [21] and Turni et al. [22].

PCRs were carried out in a final volume of 50 µL containing 5 µL of DNA template, 2.5 mM MgCl_2_, 200 µM of each deoxynucleotide triphosphate (dNTP), 20 pmol of each primer, 1 U of HOT FIREPol^®^ Taq DNA Polymerase (Solis BioDyne, Tartu, Estonia), 1× PCR buffer, and Milli-Q^®^ water to volume. Amplification products were resolved by electrophoresis on 1.5% agarose gels stained with BlueGreen^®^ (LGC Biotecnologia, Cotia, SP, Brazil). Bands were visualized using a Gel Doc XR imaging system (Bio-Rad, Munich, Germany) and compared with a 100 bp DNA ladder (New England BioLabs, Ipswich, MA, USA).

### 2.4. Antimicrobial Resistance Profiling

Minimum inhibitory concentrations (MICs) were determined by broth microdilution [23] using the BOPO6F Sensititre^®^ panel (TREK Diagnostic Systems/Thermo Fisher Scientific, Waltham, MA, USA). *A. pleuropneumoniae* ATCC 27090 and *Staphylococcus aureus* ATCC 23213 were included as quality-control strains. The following antimicrobials were tested: ampicillin (AMP), clindamycin (CLI), chlortetracycline (CTET), danofloxacin (DANO), enrofloxacin (ENRO), florfenicol (FFN), gentamicin (GEN), neomycin (NEO), oxytetracycline (OXY), penicillin (PEN), sulfadimethoxine (SDM), spectinomycin (SPE), trimethoprim/sulfamethoxazole (SXT), tiamulin (TIA), tilmicosin (TIL), tulathromycin (TUL), tylosin (TYLT), and ceftiofur (TIO).

The bacterial inoculum was prepared from a pure culture of *A. pleuropneumoniae* grown in BHI broth supplemented with NAD and fetal bovine serum and incubated at 37 °C. Culture turbidity was adjusted in sterile 0.9% saline using a spectrophotometer at 600 nm to an approximate concentration of 1 × 10^8^ CFU/mL. Subsequently, 50 µL of the adjusted suspension was diluted in 10 mL of Mueller–Hinton Fastidious broth supplemented with yeast extract (MHF-Y; Difco, Sparks, MD, USA) to obtain a final inoculum of 5 × 10^5^ CFU/mL. Then, 50 µL of this suspension was dispensed into each well of a 96-well BOPO6F Sensititre^®^ microplate (Thermo Fisher Scientific, Waltham, MA, USA). Plates were sealed with an adhesive film and incubated at 37 °C under microaerophilic conditions. MICs were read after 24 h.

MIC endpoints were determined visually as the lowest antimicrobial concentration showing no visible growth (no button formation). Isolates were categorized as susceptible, intermediate, or resistant according to the Clinical and Laboratory Standards Institute (CLSI) guidelines (VET01S, 7th ed.) [23] as described in Table 1.

### 2.5. Molecular Genotyping

#### 2.5.1. Single-Enzyme Amplified Fragment Length Polymorphism (SE-AFLP)

All strains were genotyped by single-enzyme amplified fragment length polymorphism (SE-AFLP) using HindIII (New England BioLabs, Ipswich, MA, USA), according to the protocol described by McLauchlin et al. [24]. Amplicons were separated on 2% agarose gels for 4 h at 90 V, stained with BlueGreen^®^ (LGC Biotecnologia, Cotia, SP, Brazil), and compared with a 100 bp DNA ladder (New England BioLabs, Ipswich, MA, USA).

#### 2.5.2. Pulsed-Field Gel Electrophoresis (PFGE)

For PFGE, an aliquot of bacterial culture standardized to 1 × 10^9^ CFU/mL was mixed with low-melting-point agarose (SeaKem^®^ Gold; Cambrex Bio Science, Rockland, ME, USA) and dispensed into plug molds. Agarose plugs were subjected to in situ lysis and stored in Tris–EDTA buffer until use.

A portion of each plug was digested with ApaI (New England BioLabs, Ipswich, MA, USA) and loaded onto a 1% SeaKem^®^ Gold agarose gel. Electrophoresis was performed using a CHEF-DR III system (Bio-Rad Laboratories, Hercules, CA, USA) for 24 h at 6 V/cm, with a fixed angle of 120°, initial pulse time of 5 s, and final pulse time of 20 s, in 0.5× TBE buffer maintained at 14 °C. Gels were stained for 40 min with SYBR Safe^®^ (Invitrogen, Carlsbad, CA, USA), and DNA fragments were visualized under UV illumination using a Gel Doc XR^®^ imaging system (Bio-Rad Laboratories, Hercules, CA, USA). Band sizes were estimated using a lambda DNA PFGE marker (New England BioLabs, Ipswich, MA, USA).

### 2.6. Statistical Analysis

Frequency distributions of serotypes and antimicrobial resistance profiles were analyzed using SPSS v16.0 (SPSS Inc., Chicago, IL, USA). MIC_50_ and MIC_90_ values were determined as described by Schwarz et al. [25]. Multidrug resistance (MDR) was defined according to Magiorakos et al. [26] as resistance to at least one antimicrobial agent in three or more antimicrobial classes.

Cluster analyses of SE-AFLP and PFGE profiles were performed using BioNumerics v7.6 (Applied Maths NV, Sint-Martens-Latem, Belgium). Dendrograms were generated using the Dice similarity coefficient and the unweighted pair group method with arithmetic mean (UPGMA). For SE-AFLP, a 90% similarity cutoff was applied to define clusters [26]. For PFGE, pulsotypes were distinguished based on a minimum difference of four bands between strains [27].

## 3. Results

### 3.1. Molecular Identification and Serotyping

All 85 strains were confirmed as *A. pleuropneumoniae* by PCR. Molecular serotyping yielded the following frequencies: serotype 5, 38.8% (33/85); serotype 10, 29.4% (25/85); serotype 7, 5.9% (5/85); serotype 8, 5.9% (5/85); and serotype 6, 3.5% (3/85). Fourteen strains (16.5%) were classified as non-typable.

**Figure 1:**
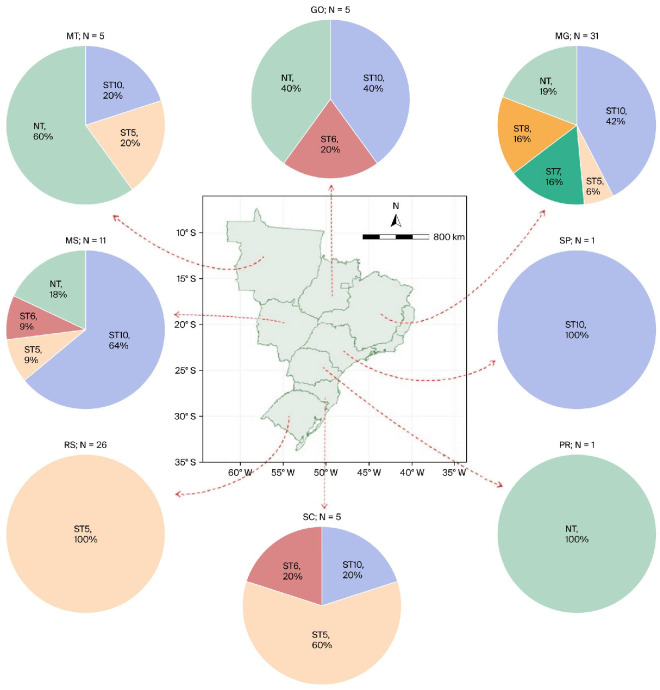
Distribution of A. pleuropneumoniae serotypes according to the Brazilian state of origin of the isolates. Pie charts show the relative frequency of serotypes within each state, NT, non-typable.

The serotype distribution across Brazilian states is shown in Figure 1. Minas Gerais (*n* = 31) included strains of serotypes 5, 7, 8, and 10, as well as non-typable strains. Rio Grande do Sul (*n* = 26) included only serotype 5 strains. Mato Grosso do Sul (*n* = 11) included serotypes 5, 6, and 10, as well as non-typable strains. Mato Grosso (*n* = 5) included serotypes 5 and 10 and non-typable strains. Goiás (*n* = 5) included serotypes 6 and 10 and non-typable strains. Santa Catarina (*n* = 5) included serotypes 5, 6, and 10.

### 3.2. Antimicrobial Resistance Profiling

All strains were susceptible to ceftiofur, gentamicin, tulathromycin, spectinomycin, and tilmicosin. Low resistance rates were observed for florfenicol (1/85, 1.2%), neomycin (1/85, 1.2%), trimethoprim/sulfamethoxazole (2/85, 2.3%), tiamulin (5/85, 5.9%), ampicillin (6/85, 7.1%), enrofloxacin (8/85, 9.4%), danofloxacin (12/85, 14.1%), and penicillin (22/85, 25.9%). In contrast, high resistance rates were detected for clindamycin (77/85, 90.6%), chlortetracycline (57/85, 67.1%), oxytetracycline (57/85, 67.1%), sulfadimethoxine (44/85, 51.8%), and tylosin (84/85, 98.8%) (Table 2). Overall, 55.2% (47/85) of the strains were classified as multidrug-resistant. Most MDR strains (44/85, 51.8%) were resistant to three or four antimicrobial classes, whereas only three strains were resistant to five or more classes.

### 3.3. Molecular Genotyping

#### 3.3.1. Single-Enzyme Amplified Fragment Length Polymorphism (SE-AFLP)

**Figure 2:**
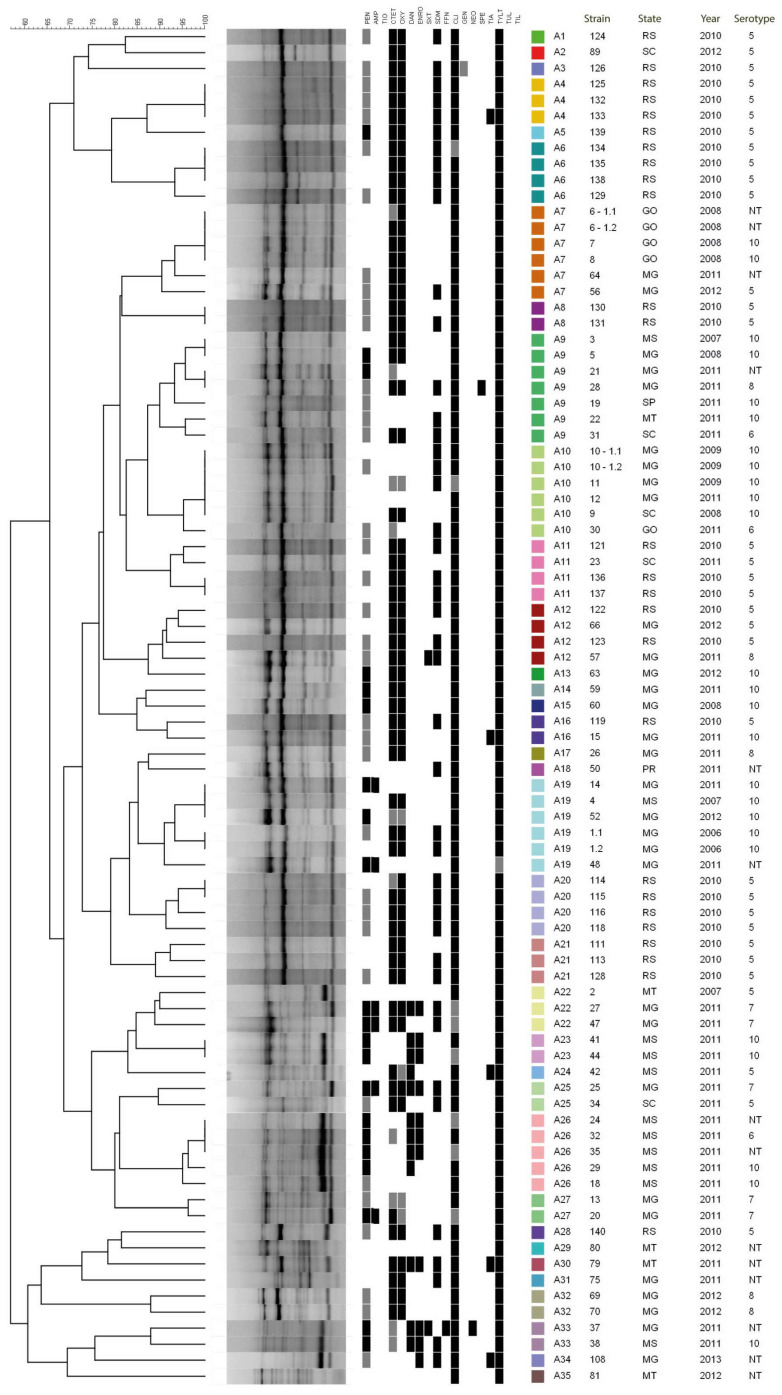
SE-AFLP dendrogram of *A. pleuropneumoniae* strains, annotated with epidemiological data and antimicrobial resistance profiles. Colored boxes adjacent to each strain identify the SE-AFLP profiles.

SE-AFLP genotyping identified 35 profiles (A1–A35). Cluster analysis generated the dendrogram shown in Figure 2. Two main groups were distinguished at <65% genetic similarity: a smaller group comprising 10 strains assigned to eight profiles (A28–A35), representing more divergent genotypes, and a larger group comprising the remaining 75 strains, which formed internal clusters from 70% similarity onward. Overall, a modest tendency for strains to cluster by geographic origin and serotype was observed (e.g., profiles A4, A6, A20, A21, and A23). However, several profiles also included strains from different origins and serotypes (e.g., A7, A9, and A26). The discriminatory index (D) for SE-AFLP was 0.97.

#### 3.3.2. Pulsed-Field Gel Electrophoresis (PFGE)

**Figure 3:**
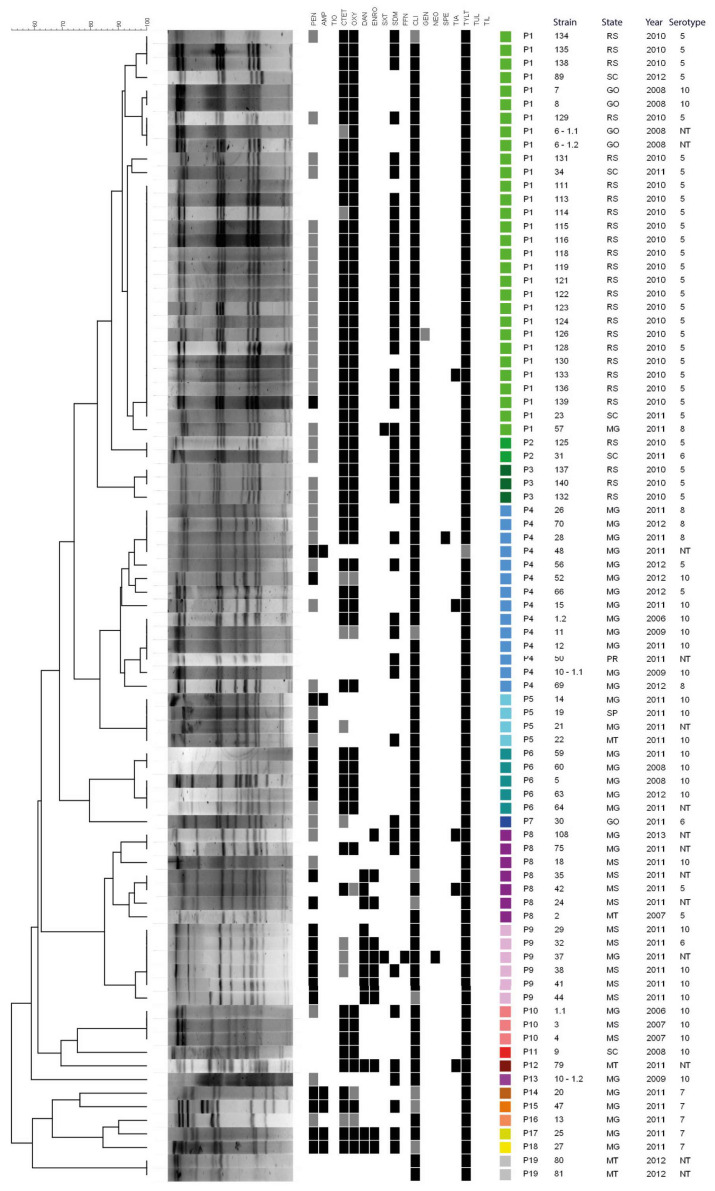
Dendrogram generated from PFGE analysis of *A. pleuropneumoniae* strains digested with the ApaI restriction enzyme, with corresponding epidemiological information and antimicrobial resistance profiles. Colored boxes adjacent to each strain identify the PFGE profiles.

PFGE identified 19 pulsotypes (P1–P19) showing >55% genetic similarity. The dendrogram (Figure 3) revealed two main groups: a smaller group comprising seven strains assigned to six pulsotypes (P14–P19), representing more divergent genotypes and consisting mostly of serotype 7 strains; and a larger group comprising the remaining 78 strains, distributed among 13 pulsotypes. Within the latter, pulsotype P1 was predominant, including 30 strains collected in different years from four states and mainly classified as serotype 5. Although some pulsotypes (e.g., P5 and P10) grouped strains of the same serotype from different geographic origins, no clear association was observed between PFGE profiles and origin, serotype, or antimicrobial resistance patterns. The discriminatory index (D) for PFGE was 0.84.

## 4. Discussion

This study evaluated the distribution of *A. pleuropneumoniae* across Brazilian states based on serotype, antimicrobial resistance patterns, and SE-AFLP and PFGE profiles. Serotype 5 was the most prevalent, followed by serotype 10, and a high level of genetic heterogeneity was observed among the strains as assessed by both AFLP and PFGE. Our findings reinforce concerns regarding the emergence of multidrug-resistant (MDR) strains, as 55% of the isolates showed resistance to more than three antimicrobial classes.

In Brazil, only serotypes 1 to 15 have been reported, with no descriptions of serovars 16 to 19 [28,29,30,31,32]. The most prevalent serotypes were previously reported as 5, 3, 6, and 10 [29] and, more recently, as 8, 7, and 5 [32]. These differences may reflect variation by region, year of isolation, and sample type (e.g., necropsy samples, nasal swabs, or slaughterhouse collections). In the present study, serotypes 5 and 10 accounted for more than 65% of the isolates.

Kuchiishi et al. [32] analyzed lung samples obtained from slaughterhouses (57%) and necropsies (43%), with most samples collected in Rio Grande do Sul (RS; 30%), Santa Catarina (SC; 28%), and Minas Gerais (MG; 12%). In our study, only lung samples from necropsies were included, and MG contributed the largest proportion of isolates, followed by RS and Mato Grosso do Sul (MS) (36.5%, 30.6%, and 12.9%, respectively). Serotype 10 was highly prevalent in MG and MS. In contrast, serotype 10 was not detected in RS, consistent with the findings of Kuchiishi et al. [32].

Despite differences among Brazilian studies, the emergence of serotypes 7 and 8 in Brazil is evident. In the present study, these serotypes were detected only in samples from Minas Gerais (MG), whereas another survey reported their occurrence in multiple states [32]. Serotype 5 was the most prevalent in both studies, suggesting a strong potential for persistence and dissemination over time and across different regions.

The global epidemiology of *A. pleuropneumoniae* shows marked regional differences in serotype distribution and antimicrobial resistance. In Asia, serotype 2 predominates in Japan (71.2%) [33]. In Taiwan, 71.4% of isolates are multidrug resistant, particularly to tetracyclines and aminoglycosides [34]. Similarly, in North Korea, approximately 70% of isolates belong to serotypes 5 and 1 [35]. In Europe, serotype distribution is also heterogeneous. Serotype 2 predominates in Poland (65.5%) [36], whereas in Hungary approximately 55% of isolates belong to serotypes 2 and 13 [30,37]. In contrast, in England and Wales, serotype 8 accounts for more than 70% of isolates across different time periods, while serotype 5 is not considered epidemiologically relevant [38]. Additionally, studies from Italy and Spain have reported the co-circulation of multiple serotypes, including 9/11, 2, 4, and 5 [14,39]. Increasing antimicrobial resistance has also been documented, including high resistance to tetracycline (up to 53%) and the emergence of multidrug-resistant strains, including isolates resistant to ≥7 antimicrobial classes [14,36]. In Africa, long-term data from South Africa indicate a predominance of serotype 7 (22.7%), followed by serotype 5, with temporal fluctuations observed over several decades [40]. In the Americas, heterogeneous patterns have also been reported. In Canada, serotypes 5 and 7 predominated from 2011 to 2014 and remained the most frequently isolated during 2015–2020, with an increasing predominance of serotype 7 [41]. In Argentina, serotype 1 accounts for 66.7% of isolates [42]. Taken together, these findings highlight the marked geographic variability in the epidemiology of *A. pleuropneumoniae* and underscore the importance of regional baseline studies across different continents.

The genotypic characterization of the studied strains by SE-AFLP revealed a high diversity of profiles (*n* = 36), which in some cases clustered strains according to serotype or geographic origin, with a discriminatory index of 0.97. To our knowledge, this is the first report describing the application of SE-AFLP to *A. pleuropneumoniae* strains. Kokotovic and Angen [10] described the use of AFLP in *A. pleuropneumoniae* using the restriction enzymes EcoRI and BspDI. The use of two restriction enzymes considerably increases the number of bands generated and generally requires automated systems for band detection and analysis. In that study, the authors reported a discriminatory index of 0.96; however, unlike the present study, all tested strains clustered according to serotype.

Analysis of the strains by PFGE separated the isolates into 19 profiles, showing a stronger association between the resulting clusters and both serotype and geographic origin; however, the discriminatory index was lower (0.84). Chevallier et al. [43] evaluated 44 strains of serotypes 1 and 5 and observed perfect clustering by serotype using ApaI, the enzyme also used in the present study. Yoo et al. [13] analyzed 102 *A. pleuropneumoniae* strains of different serotypes and years of isolation and reported high variability among PFGE profiles, with no significant correlation with serotype or year of isolation. Consistent with these findings, our SE-AFLP and PFGE analyses indicated high genetic variability, even among isolates from the same state and serotype. Moreover, polymorphism analysis of the *apxIA* gene (sequencing) in *A. pleuropneumoniae* serovar 5 identified 14 different haplotypes among 60 strains, revealing high genetic variance that may contribute to immune evasion [44].

All tested *A. pleuropneumoniae* strains were susceptible to ceftiofur, gentamicin, tilmicosin, and tulathromycin. Low resistance rates (<10%) were observed for spectinomycin, neomycin, florfenicol, ampicillin, and tiamulin. However, a study in Romania reported higher resistance to gentamicin [45]. In the present study, we observed emerging resistance to enrofloxacin and danofloxacin. Approximately one quarter of the strains were resistant to penicillin, and more than half were resistant to chlortetracycline, oxytetracycline, clindamycin, and tylosin. Similarly, other studies have reported high resistance to sulfonamides, tetracyclines, penicillin, and third-generation quinolones [12,14,15,16].

In Brazil, antimicrobial susceptibility data for *A. pleuropneumoniae* are still limited and are predominantly based on disk diffusion assays rather than MIC determination. Previous studies have reported that all evaluated strains carried genes associated with tetracycline resistance [17], and phenotypic testing by disk diffusion showed higher resistance rates to tetracycline and erythromycin, with approximately 12% of strains resistant to enrofloxacin [18]. However, these studies included a relatively small number of isolates (21 and 17, respectively) and relied on different methodological approaches, including PCR detection of resistance genes and phenotypic assays. This scenario highlights the scarcity of MIC-based data and limits direct comparisons, reinforcing the need for standardized susceptibility studies.

In line with this, although standardized therapeutic guidelines for *A. pleuropneumoniae* are limited in Brazil, available evidence indicates that ceftiofur and florfenicol—considered among the most effective therapeutic options under field conditions—together with macrolides such as tildipirosin, tilmicosin, and tulathromycin, exhibit high in vitro activity against *A. pleuropneumoniae* isolates, supporting their use in clinical settings [18]. Our results reinforce this scenario, showing complete susceptibility to ceftiofur (0% resistance) and high activity of florfenicol (1.2% resistance), as well as the absence of resistance to tilmicosin and tulathromycin. In contrast, high resistance rates were observed for tetracyclines (67.1%), tylosin (98.8%), and clindamycin (90.6%), indicating their limited usefulness for empirical therapy. From a practical perspective, ceftiofur and florfenicol emerge as the most reliable first-line options, whereas macrolides may be used strategically, further reinforcing the importance of AST-guided therapy [18,23].

This study provides a detailed characterization of the minimum inhibitory concentration (MIC) profile of *A. pleuropneumoniae* strains isolated in Brazil. A high proportion of multidrug-resistant (MDR) strains was identified (55%), representing one of the highest rates reported to date. In contrast, previous studies have described MDR prevalences of approximately 33% [13,14]. The elevated MDR rate observed in the present study may be associated with the extensive use of antimicrobials in Brazilian swine production systems. According to Dutra et al. [46], the median lifetime exposure of Brazilian pigs to antimicrobials was 73.7%, with a median of seven different drugs used.

Concerns regarding antimicrobial resistance should be emphasized, as it reduces the number of effective therapeutic options available for both humans and animals. The emergence of quinolone-resistant strains is particularly concerning because this class is categorized by the World Health Organization as “highest priority critically important antimicrobials” (WHO, 2019) [47]. Knowledge of prevalent serotypes, resistance patterns, and genotypic profiles is essential to define the epidemiology of *A. pleuropneumoniae* and may support more targeted prevention and treatment protocols, thereby reducing the use of ineffective drugs in Brazil.

## 5. Conclusions

This genotypic and phenotypic assessment of *A. pleuropneumoniae* strains from different Brazilian states revealed substantial pathogen diversity and indicated that PFGE correlated more closely with epidemiological data. Despite the widespread use of antimicrobials in Brazilian pig production for the prevention and control of bacterial diseases, most *A. pleuropneumoniae* isolates remained susceptible to beta-lactams, tilmicosin, tulathromycin, florfenicol, spectinomycin, enrofloxacin, danofloxacin, and neomycin. Nevertheless, a high proportion of multidrug-resistant strains was observed. Further studies are needed to monitor the dynamics of *A. pleuropneumoniae* infection, the emergence of new dominant serotypes, and trends in antimicrobial resistance.

## Figures and Tables

**Table 1:** Criteria for analyzing MIC results (breakpoints).

Antimicrobial Agents	MIC (µg/mL)		
	Sensitive	Intermediate	Resistant
Ampicillin	≤0.5	1	≥2
Ceftiofur	≤2	4	≥8
Penicillin	≤0.25	0.5	1
Chlortetracycline	≤0.5	1	≥2
Oxytetracycline	≤0.5	1	≥2
Florfenicol	≤2	-	≥8
Danofloxacin	≤0.25	-	-
Enrofloxacin	≤0.25	-	≥1
Gentamicin	≤2	4	≥8
Neomycin	≤8	-	-
Spectinomycin	≤32	-	≥128
Clindamycin	≤0.5	1–2	≥4
Tylosin	≤1	2–4	>4
Tiamulin	≤16	-	≥32
Tilmicosin	≤16	-	≥32
Tulathromycin	≤64	-	-
Sulfadimethoxine	≤256	-	≥512
Trimethoprim/Sulfamethoxazole	≤2/38	-	≥4/76

MIC: minimum inhibitory concentration; -: no interpretive breakpoint available.

**Table 2:** Distribution of minimum inhibitory concentrations (MICs), MIC_50_, MIC_90_, and resistance rates among 85 *A. pleuropneumoniae* strains.

Antimicrobial	Number of Strains with MIC (µg/mL)										MIC_50_ (µg/mL)	MIC_90_ (µg/mL)	Resistant %
	0.12	0.25	0.5	1	2	4	8	16	32	64			
Penicillin	3	24	36	16	0	1	0	5	-	-	0.5	1	25.9
Ampicillin	-	75	4	0	0	0	1	0	5	-	0.25	0.5	7.1
Ceftiofur	-	85	0	0	0	0	0	-	-	-	0.25	0.25	0.0
Chlortetracycline	-	-	18	10	26	21	10	-	-	-	2	8	67.1
Oxytetracycline	-	-	23	5	0	1	12	44	-	-	16	16	67.1
Danofloxacin	66	7	2	2	8	-	-	-	-	-	0.12	1	14.1
Enrofloxacin	67	6	4	0	0	8	-	-	-	-	0.12	0.5	12.9
Florfenicol	-	20	60	4	0	0	1	-	-	-	0.5	0.5	1.2
Spectinomycin	-	-	-	-	-	-	0	5	79	1	32	32	0.0
Gentamicin	-	-	23	2	59	1	0	0	0	0	2	2	0.0
Neomycin	-	-	-	-	-	76	8	1	0	0	4	8	1.2
Clindamycin	-	0	0	0	8	28	46	2	1	0	8	8	90.6
Tylosin	-	-	0	0	0	1	1	7	56	20	32	64	98.8
Tilmicosin	-	-	-	-	-	22	58	5	0	0	8	8	0.0
Tulathromycin	-	-	-	0	7	9	50	19	0	0	8	16	0.0
Tiamulin	-	-	0	0	1	0	11	68	5	0	16	16	5.9
Antimicrobial	N° of isolates with MIC of (µg/mL)												
Sulfadimethoxine	≤256					>256					MIC_50_	MIC_90_	Resistant
	41					44					>256	>256	51.7
Trimethoprim/ sulfamethoxazole	≤2/38					>2/38					MIC_50_	MIC_90_	Resistant
	83					2					≤2/38	≤2/38	2.35

MIC: minimum inhibitory concentration; Res: resistance rate; “-“: dilution not included in the antimicrobial panel or value not applicable.

## Data Availability

The original contributions presented in this study are included in the article. Further inquiries can be directed to the corresponding author.
